# Fixel-based analysis reveals whole-brain white matter microstructural alterations in axial spondyloarthritis

**DOI:** 10.1038/s41598-026-45157-1

**Published:** 2026-03-20

**Authors:** Wei Wang, Yang Yang, Yang Xue, Ye Wang, Yamin Luo, Yang Liu, Fan Lu, Guiping Shen, Yi Xiao, Zhongyu Liu, Fei Xiong

**Affiliations:** 1 Department of Radiology, The 989th Hospital of the Joint Logistic Support Force of PLA, Luoyang, Henan Province China; 2 Department of Radiology, General Hospital of Central Theater Command, No. 627 Wuluo Road, Wuchang District, Wuhan, Hubei Province China; 3 Department of General Surgery, The 989th Hospital of the Joint Logistic Support Force of PLA, Luoyang, Henan Province China; 4 Department of Radiology, Changzheng Hospital affiliated to Navy Military Medical University, Shanghai, China; 5 Medical Support Training Center, The 989th Hospital of the Joint Logistic Support Force of PLA, No. 2 Huaxia West Road, Luoyang, 471031 Henan Province China

**Keywords:** Axial spondyloarthritis, White matter, Diffusion MRI, Fixel-based analysis, Neuroinflammation, Structural remodeling, Biomarkers, Diseases, Medical research, Neurology, Neuroscience, Rheumatology

## Abstract

Axial spondyloarthritis (axSpA) is a chronic inflammatory disorder that may influence the central nervous system through systemic inflammation. While functional brain alterations have been reported, white matter microstructural changes remain poorly understood. This study investigated white matter alterations in axSpA using fixel-based analysis (FBA). Thirty-nine axSpA patients (mean age 28.5 ± 6.3 years) and 41 healthy controls (29.1 ± 5.8 years) underwent diffusion MRI. FBA assessed fiber density (FD), fiber-bundle cross-section (FC), and combined fiber density and cross-section (FDC) across the whole brain. Group differences were analyzed controlling for age, sex, and education. Clinical correlations were assessed with False Discovery Rate (FDR) correction. Compared to controls, axSpA patients showed significantly increased FC in the left external capsule (EC) and increased FDC in the right EC and right uncinate fasciculus (UF) (family-wise error corrected *P* < 0.05). No significant FD alterations were observed. In exploratory analyses, left EC FC showed a nominal positive correlation with physical function scores (Rho = 0.21, uncorrected *P* = 0.037), but this association did not survive FDR correction (adjusted *P* > 0.05). axSpA involves asymmetric white matter remodeling with increased bundle cross-section in corticostriatal and limbic pathways. These findings likely reflect active neuroinflammatory processes—such as maladaptive structural remodeling or compensatory hypertrophy—likely representing a distinct mechanism from the atrophy observed in later stages of neurodegeneration. FBA provides sensitive metrics for detecting these subtle macrostructural alterations in axSpA.

**Registry**: chictr.org.cn, TRN: ChiCTR2500111024, Registration date: 23rd October 2025.

**Supplementary Information:**

The online version contains supplementary material available at 10.1038/s41598-026-45157-1.

## Introduction

Axial spondyloarthritis (axSpA) is a chronic inflammatory disorder primarily affecting the axial skeleton and sacroiliac joints. Recent evidence suggests that axSpA may influence the central nervous system through systemic inflammation and neuro-immune interactions^1,2^. This has led to increased interest in understanding brain alterations in axSpA patients.

Neuroimaging studies have revealed brain changes in axSpA that extend beyond pain processing circuits. Patients with axSpA show altered resting-state brain networks, particularly in attention and sensory salience networks^3^. These changes correlate with disease-related fatigue, a prominent symptom affecting up to 65% of patients^4^. Additionally, axSpA patients have high rates of psychiatric comorbidities, with depression occurring in 11–64% and anxiety in 13–47% of cases^5,6^. Neuroimaging studies have identified aberrant connectivity in the default mode network and frontoparietal networks in these patients^7^.

The symptom profile of axSpA includes chronic pain, persistent fatigue, and elevated psychological distress. These symptoms suggest complex brain network alterations involving sensory, attention, and emotional regulation circuits^8,9^. While most neuroimaging studies in axSpA have focused on functional connectivity and gray matter alterations, emerging evidence from related inflammatory conditions suggests that white matter microstructure may also be affected. Studies in rheumatoid arthritis have demonstrated white matter alterations using diffusion tensor imaging (DTI), with fractional anisotropy value correlating with fatigue severity^10,11^. Similarly, patients with systemic lupus erythematosus show longitudinal white matter microstructural alterations that parallel disease progression^12^. However, direct investigation of white matter integrity in axSpA remains limited.

Traditional DTI approaches have inherent limitations, particularly in regions with crossing fibers, which comprise up to 90% of white matter voxels^13^. These limitations may explain the scarcity of white matter studies in axSpA, as conventional DTI metrics can be confounded in complex fiber configurations. Furthermore, conventional DTI metrics often conflate distinct pathological processes. For instance, decreased fractional anisotropy (FA) can result from demyelination, axonal loss, or complex fiber geometry. In the context of inflammatory diseases, alterations in white matter volume or density might reflect diverse mechanisms, ranging from adaptive plasticity to neuroinflammatory processes such as reactive astrogliosis or compensatory structural remodeling. Fixel-based analysis (FBA) overcomes these constraints by examining specific fiber populations within each voxel^14,15^. FBA separately quantifies fiber density (FD) and fiber bundle cross-section (FC), providing a more specific characterization of white matter remodeling that is essential for distinguishing between atrophy and volumetric expansion potentially driven by inflammation^16^.

The clinical relevance of white matter investigation in axSpA is supported by evidence linking peripheral inflammation to central neuroinflammation. Chronic elevation of inflammatory mediators in axSpA, such as TNF-α and IL-17 may impact white matter through blood-brain barrier disruption and microglial activation. The high prevalence of neuropsychiatric symptoms in axSpA suggests that white matter alterations may contribute to disease-related disability^17,18^. Despite accumulating functional connectivity findings and evidence from related inflammatory conditions, no study has comprehensively characterized white matter microstructural alterations in axSpA using advanced diffusion techniques. FBA methodology may reveal fiber-specific changes underlying the complex symptom profile in axSpA patients.

This study aimed to investigate whole-brain white matter microstructural alterations in axSpA patients compared to healthy controls using FBA. We hypothesized that FBA would reveal white matter microstructural alterations in axSpA patients, and that these alterations may be associated with clinical disease characteristics and functional outcomes.

## Results

### Demographic and clinical characteristics

This study included 39 axSpA patients and 41 HC participants, well-matched for age, sex distribution, and body mass index (all *P* > 0.05). Significant differences were observed in sociodemographic variables. Marital status differed between groups (axSpA vs. HC: 38.5% vs. 65.9%, *P* = 0.021). Educational attainment showed marked disparities (*P* < 0.001), with 85.4% of HC participants having undergraduate or higher education versus only 35.9% of axSpA patients.

Health-related quality of life measures revealed substantial impairments in the axSpA group. Physical function scores were significantly lower in axSpA patients [median 50.00, IQR 20.00] compared to HC [median 92.50, IQR 12.50] (*P* < 0.001). Physical role functioning was severely compromised in axSpA patients [median 25.00, IQR 54.13] versus HC [median 100.00, IQR 0.00] (*P* < 0.001). Bodily pain scores demonstrated significant impairment in the patient group [median 31.00, IQR 17.00] compared to HC [median 84.00, IQR 18.00] (*P* < 0.001).

Clinical characteristics of axSpA patients included median duration of low back pain of 31 months [IQR 58.00], median medication duration of 6.00 months [IQR 47.00], and HLA-B27 positivity in 61.5% of patients. Inflammatory markers showed elevated levels with median ESR of 7.00 mm/h [IQR 7.50] and median CRP of 5.00 mg/L [IQR 7.85]. Details were shown in Table 1 .

**Table 1:** Comparison of demographic and clinical data.

Characteristics	axSpA (*n* = 39)	HC (*n* = 41)	Statistic value	*P*	Effect size
Age (years)	24.00 (8.50)	25.00 (3.00)	− 0.37	0.713	0.041
Sex (male)	33 (84.6%)	28 (68.3%)	2.11	0.146	0.162
BMI (Kg/m^2^)	27.85 (10.39)	28.79 (11.43)	− 0.383	0.703	0.086
Married	13 (33.3%)	5 (12.2%)	5.121	0.021	0.253
Education			25.62	0.024	0.566
Junior High School	2 (5.1%)	0 (0.0%)			
Senior High School	7 (17.9%)	0 (0.0%)			
Associate Degree	13 (33.3%)	4 (9.8%)			
Bachelor’s Degree	15 (38.5%)	21 (51.2%)			
Master’s Degree or above	2 (5.1%)	16 (39.0%)			
Duration of Low Back Pain (months)	31.00 (58.00)	NA	NA	NA	NA
Medication Duration (months)	6.00 (47.00)	NA	NA	NA	NA
HLA-B27 (ng/µL)	156.96 (11.84)	NA	NA	NA	NA
ESR (mm/h)	7.00 (7.50)	NA	NA	NA	NA
CRP (mg/L)	5.00 (7.82)	NA	NA	NA	NA
Clinical & neuropsychiatric assessments					
Physical Function	90.00 (30.00)	100.00 (0.00)	− 5.27	< 0.001	0.59
Physical Role Functioning	50.00 (100.00)	100.00 (0.00)	− 4.22	< 0.001	0.471
Bodily Pain	50.00 (25.00)	100.00 (20.00)	− 5.23	< 0.001	0.585
General Health	60.00 (32.50)	50.00 (10.00)	0.75	0.453	0.084
Vitality	70.00 (30.00)	85.00 (10.00)	− 3.91	< 0.001	0.437
Social Functioning	88.89 (22.22)	88.89 (0.00)	− 0.75	0.436	0.083
Emotional Role Functioning	100.00 (100.00)	100.00 (0.00)	− 2.64	< 0.001	0.295
Mental Health	76.00 (22.00)	92.00 (12.00)	− 4.31	< 0.001	0.482
PSQI	47.74 (15.42)	43.98 (10.63)	2.08	0.036	0.232
MFI-20	2.00 (3.55)	0.00 (0.00)	1.27	0.21	0.286
Night Pain	1.60 (3.85)	0.00 (0.00)	6.31	< 0.001	0.706
Overall Back Pain	0.90 (2.40)	0.00 (0.00)	4.74	< 0.001	0.529
BASDAI Activity Index	1.32 (1.76)	NA	NA	NA	NA
BASFI Function Index	0.34 (1.65)	NA	NA	NA	NA
BASMI Metrology Index	1.00 (1.00)	NA	NA	NA	NA
SAS	54.00 (6.00)	52.00 (6.00)	2.55	0.01	0.285
SDS	39.50 (20.62)	36.25 (13.75)	1.52	0.129	0.17
MoCA	29.00 (2.00)	29.00 (4.00)	− 0.36	0.716	0.04

ESR: erythrocyte sedimentation rate; CRP: C-reactive protein; PSQI: Pittsburgh Sleep Quality Index; MFI-20: Multidimensional Fatigue Inventory; BASDAI: Bath Ankylosing Spondylitis Disease Activity Index; BASFI: Bath Ankylosing Spondylitis Functional Index; BASMI: Bath Ankylosing Spondylitis Metrology Index; SAS: Self-Rating Anxiety Scale; SDS: Self-Rating Depression Scale; MoCA: Montreal Cognitive Assessment.

### Fixel-based analysis results

Whole-brain FBA was performed to assess white matter microstructural alterations in axSpA patients compared to HC across three complementary metrics: FD, FC, and FDC. After controlling for age, sex and education level, axSpA patients demonstrated no significant alterations in FD across the whole brain compared to HC. However, a notable increase in FC was identified in the left EC. Additionally, increases in FDC were observed in the right EC and right UF (FWE-corrected *P* < 0.05).

**Fig. 1:**
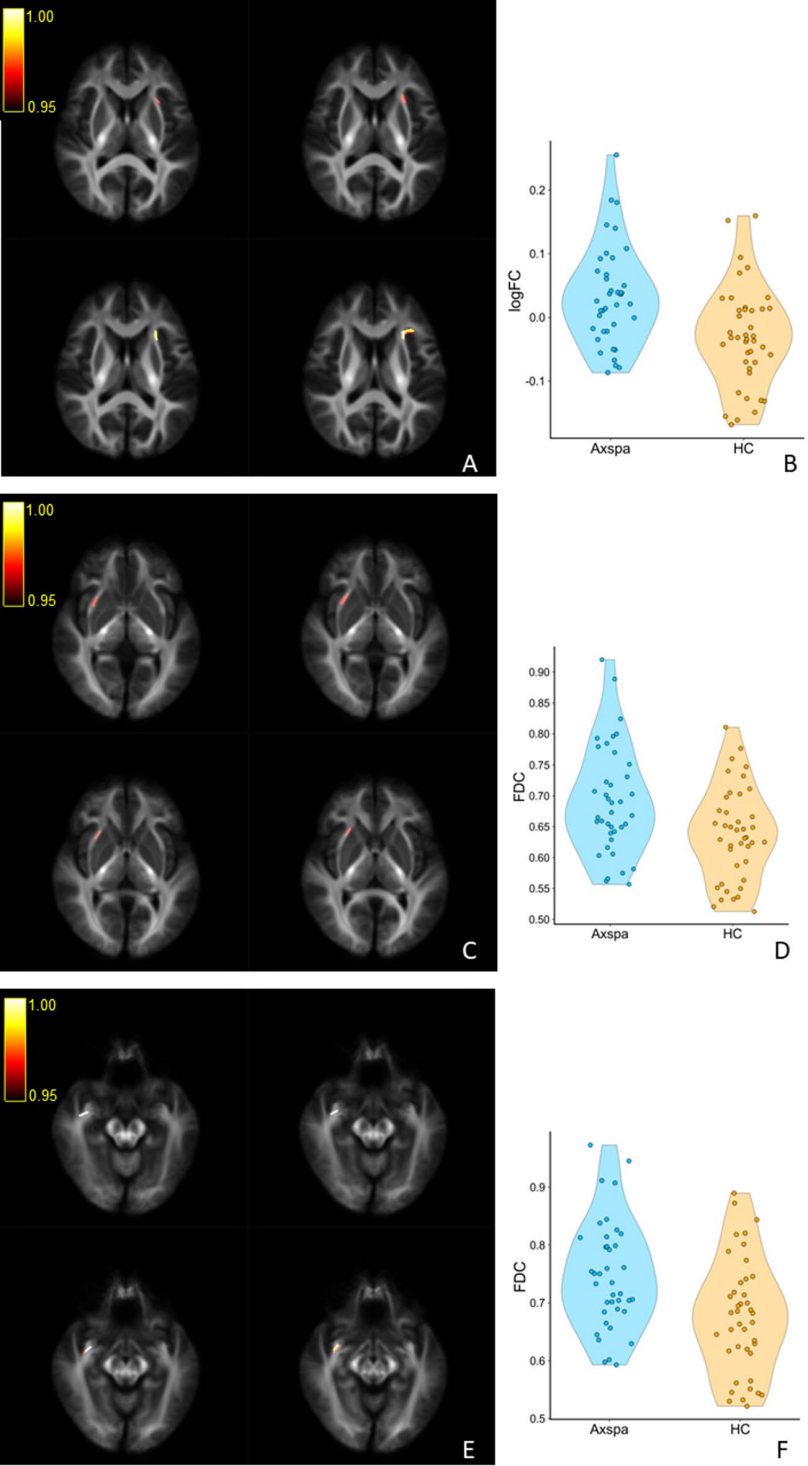
Significant between-group differences in fiber-specific measures from Fixel-Based Analysis (FBA). (**A**) A significant cluster (FWE-corrected *P* < 0.05) in the left external capsule (EC). (**B**) Violin plot of the mean logFC values for each participant within the cluster shown in (**A**). (**C**) The first significant cluster (FWE-corrected *P* < 0.05) located in the right external capsule (EC). (**D**) Violin plot of the mean FDC values for each participant within the cluster shown in (**C**). (**E**) The second significant FDC cluster (FWE-corrected *P* < 0.05) located in the right uncinate fasciculus (UF). (**F**) Violin plot of the mean FDC values for each participant within the cluster shown in (**E**).

These findings indicate that axSpA patients exhibit increased fiber-bundle cross-sectional measures in bilateral ECs and combined increases in both fiber density and cross-sectional measures in the right UF, suggesting region-specific white matter structural alterations in the patient population (Table 2; Fig. 1).

**Table 2:** Significant FBA results in axSpA patients compared to HC.

Metrics	axSpA group	HCs	Test values	*P*	Cohen’d	95% CI
	*n* = 39 (mean, SD)	*n* = 41 (mean, SD)				
logFC (left EC)	0.033 (0.077)	− 0.031 (0.078)	3.682	< 0.001	0.823	0.364–1.278
FDC (right EC)	0.691 (0.086)	0.637 (0.074)	2.975	0.004	0.665	0.213–1.114
FDC (right UF)	0.747 (0.093)	0.676 (0.098)	3.314	0.001	0.741	0.286–1.193

Generalized linear models were used for analysis, and education level as control variables. axSpA: axial spondyloarthritis; HC: healthy controls; EC: external capsule; UF: uncinate fasciculus; FC: fiber bundle cross-section; FDC: fiber density and cross-section.

### Comparisons with DTI metrics

To contextualize the FBA findings, we performed a supplementary analysis of conventional DTI metrics in the regions showing significant FBA alterations. No statistically significant differences were found between axSpA patients and HC in either FA or MD values within the left EC, right EC, or right UF (all *P* > 0.05).

### Sensitivity analysis and medication effects

To evaluate whether the observed white matter alterations were confounded by the use of anti-TNF biologics, we performed a sensitivity analysis excluding 16 patients receiving such therapy. Using generalized linear models controlling for age, sex and education level, we compared the extracted fixel metrics from the significant ROIs between the remaining untreated patients (*n* = 16) and HCs (*n* = 41). The analysis confirmed that the alterations remained statistically significant in the untreated subgroup: logFC in the left EC (*P* = 0.023), FDC in the right EC (*P* = 0.041), and FDC in the right UF (*P* = 0.019) (adjusted BH-FDR *P* < 0.05) (Supplementary Table S1). Furthermore, no significant correlations were observed between the duration of medication and these fixel metrics (all *P* > 0.05).

### Clinical significance of white matter alterations

Clinical correlations were examined between white matter alterations and clinical measures in axSpA patients using partial correlation analysis, controlling for age, sex and education level. In exploratory analyses, a nominal positive correlation was found between mean logFC values in the left EC and Physical Function scores (Rho = 0.21, *P* = 0.037). However, this association did not survive FDR correction for multiple comparisons (adjusted *P* > 0.05). No significant correlations were observed between FDC alterations in the right EC or right UF and any clinical measures, including disease duration, inflammatory markers, or quality of life scores (all adjusted *P* > 0.05).

## Discussion

This study presents the first FBA of white matter architecture in axSpA. We observed regionally specific white matter alterations: increased FC in the left EC, and increased FDC in the right EC and right UF. Importantly, these structural differences persisted after controlling for education level, suggesting that the observed effects are unlikely to be fully explained by demographic differences alone.

FBA quantifies microstructural changes (FD; reflecting intra-axonal volume of aligned fibers) and macrostructural changes (FC; representing relative fiber-bundle cross-section), along with their combination (FDC)^14,15^. The pattern of preserved FD with localized increases in FC and combined FDC metrics suggests targeted macrostructural remodeling rather than widespread microstructural axonal alterations. While we initially hypothesized that these increases might reflect adaptive neuroplasticity, alternative neuroinflammatory mechanisms must be considered. The isolated FC increase in the left EC most likely reflects macrostructural bundle expansion. In the context of chronic inflammation, the observed profile–increased FC/FDC with intact FD–is most consistent with compensatory hypertrophic remodeling or reactive astrogliosis, where an increase in net tissue volume (e.g., via axonal sprouting or glial proliferation) occurs without compromising fiber density^19^. Animal models of inflammation have demonstrated that microglial activation can lead to increased volume metrics in white matter tracts prior to the onset of atrophy^20^. Therefore, the remodeling observed here likely represent a complex interplay between potential compensatory hypertrophy and neuroinflammatory tissue expansion.

The pattern of lateralization–isolated FC increase in the left EC versus FDC increases in the right EC and right UF–indicates differential micro- and macrostructural alterations across brain regions. Anatomically, the EC contains corticostriatal and association fibers connecting frontal and subcortical regions, playing a critical role in cognitive flexibility and the regulation of motor planning^21^. The UF is a key limbic tract linking the amygdala and temporal pole with the orbitofrontal cortex, essential for emotion regulation and episodic memory^22^. The involvement of these pathways suggests that axSpA affects circuits integrating cognitive, emotional, and motor control functions, rather than primary sensory-motor transmission.

Our supplementary analysis revealed no significant differences in conventional DTI metrics within the identified regions. This finding stands in contrast to previous DTI studies in axSpA, which have reported reductions in FA indicative of microstructural impairment^23^. We propose that this discrepancy stems from two critical factors. First, regarding methodology, the EC and UF are hubs for extensive crossing fibers^24^. In these complex areas, the conventional tensor model often conflates fiber complexity with pathology, potentially masking specific bundle expansions. By using Constrained Spherical Deconvolution, FBA resolves these crossing populations, revealing structural details that DTI fails to capture. Second, regarding mechanism, the contrast between our findings and prior literature likely reflects the disease stage. While reduced FA typically indicates chronic neurodegeneration, active neuroinflammatory processes trigger reactive astrogliosis, characterized by cellular hypertrophy and proliferation^25^. These processes effectively increase tissue volume and density prior to irreversible axonal loss. Thus, our findings likely capture an active and potentially reversible phase of inflammatory remodeling, distinct from the degenerative patterns observed in other cohorts.

Neuroimaging studies in rheumatoid arthritis and systemic lupus erythematosus have reported white matter alterations associated with fatigue and cognitive impairment^10,12,26^. In our exploratory analysis, uncorrected results suggested a positive correlation between left EC FC and physical function scores (Rho = 0.21, *P* = 0.037). However, this correlation did not survive False Discovery Rate (FDR) correction (adjusted *P* > 0.05). While the uncorrected trend aligns with our hypothesis that maintained structural volume in corticostriatal pathways might support residual physical performance, the effect size was modest and did not survive rigorous statistical correction. Consequently, we interpret these associations with caution. Rather than definitive evidence of adaptive plasticity, these findings should be viewed as preliminary signals warranting validation in larger cohorts.

Despite comprehensive assessment of anxiety and depression, none of the identified white matter alterations correlated with emotional measures. This is particularly noteworthy given the anatomical significance of the UF in affective regulation. The absence of emotional correlations in our study could stem our younger patient population with shorter disease duration, where structural remodeling may precede clinically detectable dysfunction, or the need for more specialized neuropsychological assessments to detect subtle cognitive-affective changes.

Systemic inflammation disrupts blood-brain barrier function, exposing central white matter to circulating inflammatory mediators^19,27^. Cytokines elevated in axSpA (TNF-α, IL-17, IL-1β) modulate microglia and oligodendrocyte function, potentially influencing white matter integrity^28^. The IL-23/IL-17 axis is central to axSpA pathogenesis. Circulating IL-17 can compromise blood-brain barrier integrity and promote neuroinflammation^29,30^. We speculate that the observed increases in fiber cross-section might partially reflect neuroinflammatory responses driven by this systemic inflammatory milieu. Specifically, cytokine-induced BBB disruption could facilitate the influx of inflammatory mediators, leading to microglial activation and reactive remodeling^31^, which would manifest as increased bundle cross-section in FBA. Although we lacked specific cytokine data to test this directly, future studies incorporating serum or CSF levels of IL-17 are needed to validate this hypothesis.

It is also crucial to consider the potential influence of pharmacotherapy. Anti-TNF agents have been rarely linked to central nervous system demyelination events^32,33^, which would typically manifest as reduced white matter integrity (e.g., decreased fiber density). However, our study observed increased FC and FDC metrics. This directionality–reflecting structural reinforcement or expansion rather than degradation–suggests that the observed changes are unlikely to be adverse side effects of medication. This conclusion is further supported by our sensitivity analysis, where the pattern of white matter alterations persisted in the subgroup of patients not receiving biologic therapy. Nevertheless, we acknowledge that sample size limitations in subgroup analyses warrant caution. Future longitudinal studies tracking brain structural trajectories before and after the initiation of biologic therapy are essential to definitively disentangle treatment-induced neuroplasticity from disease-related pathology.

Several limitations should be considered. First, the cross-sectional design precludes causal inferences regarding whether white matter remodeling precedes or follows clinical onset; longitudinal follow-up is required to delineate the temporal trajectory. Second, although FBA provides specific metric changes, our biological interpretation of compensatory hypertrophy or neuroinflammation remains inferential without histological validation. Third, regarding sample characteristics, the strict age range (18–40 years) minimized aging-related confounds but limits generalizability to older populations. Notably, there was a significant disparity in educational attainment between groups. While we strictly controlled for education as a covariate and the observed focal alteration patterns (limbic/corticostriatal) differ from the global effects typically associated with education, residual confounding cannot be entirely ruled out. Future studies employing propensity score matching are recommended to confirm these findings. Fourth, the lack of correlation with routine inflammatory markers (ESR/CRP) suggests that specific cytokines (e.g., IL-17) may be more relevant to structural remodeling and should be quantified in future work. Finally, the acquisition slice thickness (3.0 mm) may introduce partial volume effects, necessitating high-resolution isotropic imaging for validation.

## Conclusions

This study reveals that axSpA involves asymmetric white matter remodeling characterized by increased bundle cross-section in the left EC and combined micro- and macrostructural changes in right-sided regions. While uncorrected analyses hinted at a link between left EC structure and physical function, rigorous statistical correction suggests these associations require further validation. Collectively, our findings suggest that fixel-based metrics may capture neuroplastic or compensatory processes—such as hypertrophic remodeling or bundle expansion—that are distinct from the degenerative patterns typically seen in chronic stages. These insights expand our understanding of central nervous system involvement in axSpA and underscore the potential of fixel-based metrics as sensitive biomarkers of disease impact and therapeutic response.

## Methods

### Study population

This study was approved by the Institutional Ethics Committee of General Hospital of Central Theater Command (Reference: [2025] 15–101) and conducted in accordance with the Declaration of Helsinki. Written informed consent was obtained from all participants.

A total of 85 participants were initially recruited, with 5 participants excluded due to inadequate image quality from motion artifacts or non-compliance during scanning, resulting in a final sample of 80 participants comprising 39 axSpA patients from the Department of Rheumatology and 41 healthy controls (HC) from the general population.

Inclusion criteria for axSpA patients: (1) fulfilled the 2009 Assessment of Spondyloarthritis International Society (ASAS) classification criteria for axSpA^34^; (2) aged 18–40 years; (3) right-handed according to the Edinburgh Handedness Inventory; (4) disease duration ≥ 6 months; (5) clinically stable condition for at least 4 weeks prior to study enrollment; and (6) no contraindications to MRI examination.

Inclusion criteria for healthy controls: (1) aged 18–40 years; (2) right-handed according to the Edinburgh Handedness Inventory; (3) absence of chronic pain conditions (pain duration > 3 months), inflammatory or autoimmune diseases, or current psychiatric disorders; (4) no history of neurological disorders or head trauma with loss of consciousness; (5) no history of psychoactive medication use; and (6) no contraindications to MRI examination.

Exclusion criteria for both groups: (1) pregnancy; (2) history of substance abuse; (3) major medical comorbidities affecting brain structure or function; (4) inadequate image quality due to motion artifacts; and (5) incomplete clinical or imaging data.

**Fig. 2:**
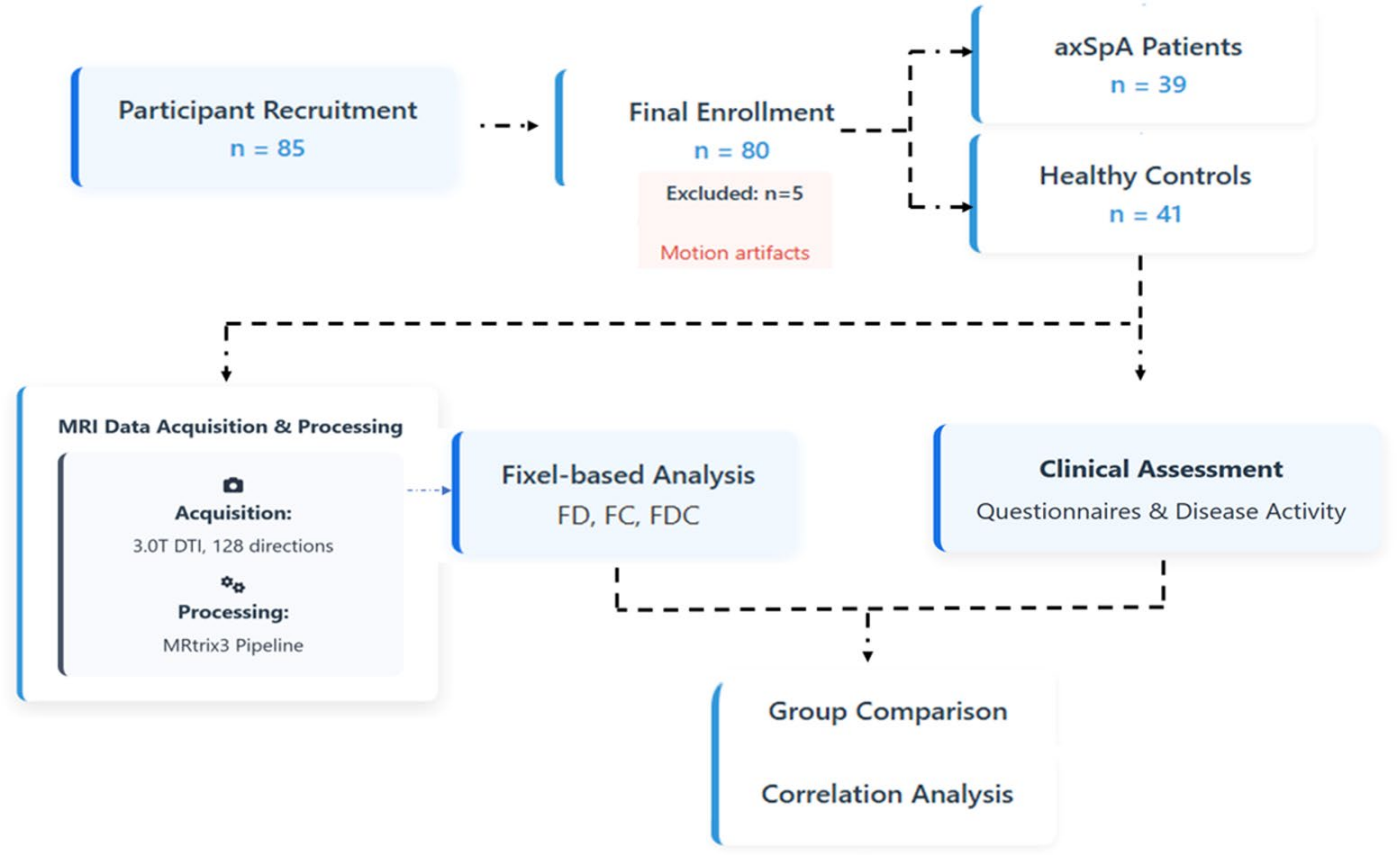
Flow chart.

Demographic data (age, sex, BMI, marital status, education) were collected from all participants. For axSpA patients, clinical parameters including disease duration, medication duration, HLA-B27 status, erythrocyte sedimentation rate (ESR), and C-reactive protein (CRP) were recorded within 24 h of the MRI. All participants completed standardized questionnaires including Short Form 36 (SF-36) health survey, Pittsburgh Sleep Quality Index (PSQI), Multidimensional Fatigue Inventory (MFI-20), Self-Rating Anxiety Scale (SAS), Generalized Anxiety Disorder-7 (GAD-7), Self-Rating Depression Scale (SDS), and Montreal Cognitive Assessment (MoCA). The axSpA patients additionally completed disease-specific assessments including Bath Ankylosing Spondylitis Disease Activity Index (BASDAI)^35^, Bath Ankylosing Spondylitis Functional Index (BASFI), Bath Ankylosing Spondylitis Metrology Index (BASMI), night pain score, and overall back pain score. The research process was shown in Figure 2.

### MRI data acquisition

All scans were performed on a 3.0T United Imaging uMR 770 system (United Imaging Healthcare, Shanghai, China) with a 32-channel head coil. Participants were positioned supine with foam padding for head stabilization.

Diffusion tensor imaging used single-shot echo-planar imaging with the following parameters: TR = 19.03ms, TE = 115.50 ms, FOV = 224 × 224 mm², acquisition matrix = 160 × 160, reconstruction matrix = 160 × 160, slice thickness = 3.0 mm (gap = 0.9 mm), 46 axial slices covering the whole brain, flip angle = 90°. The diffusion protocol included 128 directions across multiple b-values: 1 image at b = 0 s/mm², 63 images at b = 1000 s/mm², and 64 images at b = 2000 s/mm². Parallel imaging acceleration factor was 2. Total acquisition time was approximately 41 min. All images underwent immediate quality control, with repeat scanning when necessary.

To ensure the quality of the data and minimize motion artifacts, we assessed participants’ motion by calculating framewise displacement (FD_motion) as described by Power et al.^36^, which provides a metric of head motion throughout the scanning session. We calculated FD_motion for each subject and found no significant differences in motion between axSpA patients and healthy controls (*P* > 0.05). This indicates that motion-related artifacts are unlikely to have influenced our findings. Additionally, we evaluated image registration quality using metrics such as template alignment accuracy and visual inspection for artifacts.

### Data processing

Diffusion data were preprocessed using MRtrix3 (version 3.0.5)^16^, including denoising^37^, Gibbs ringing removal, eddy current and motion correction, bias field correction, and upsampling to isotropic 1.3 mm³ voxels. Fiber orientation distributions (FODs) were estimated using multi-shell, multi-tissue constrained spherical deconvolution (MSMT-CSD)^13^ with group-averaged response functions. An unbiased population template was generated from all 80 participants (39 axSpA, 41 HCs) through iterative FOD registration and averaging^38^. Individual FODs were registered to this template using nonlinear registration. Probabilistic tractography generated 20 million streamlines, filtered to 2 million using Spherical-deconvolution Informed Filtering of Tractograms (SIFT)^39^. Fixel-based metrics—FD, FC, and FDC—were computed in template space for statistical analysis^14^. Additionally, FA and MD values were computed from the diffusion tensor model fitted to the diffusion data, providing insights into the microstructural integrity of white matter.

### Statistical analysis

All statistical analyses were performed using R software (version 4.5.0) and MRtrix3 statistical functions.

Data normality was assessed using the Kolmogorov-Smirnov test. Continuous variables were compared between groups using independent t-tests for normally distributed data or Mann-Whitney U tests for non-normally distributed data. Categorical variables were analyzed using χ² tests or Fisher’s exact test as appropriate.

Between-group differences in white matter microstructure were examined using FBA^14^. A general linear model was constructed for each metric (FD, FC, FDC) with group as the main factor controlling for age, sex and education level as covariates. Statistical significance was determined using connectivity-based fixel enhancement (CFE) with non-parametric permutation testing (5,000 permutations) and family-wise error correction to control for multiple comparisons across all fixels (*P* < 0.05).

Fixels showing significant group differences were extracted for correlation analyses with clinical variables in the axSpA group. Pearson correlation coefficients were used for normally distributed variables and Spearman rank correlation for non-normally distributed variables. P-values were adjusted for multiple comparisons using the False Discovery Rate (FDR) method (Benjamini-Hochberg procedure).

Mean FA and MD values were extracted from the significant fixel-derived masks and compared between groups using general linear models controlling for age, sex and education. To assess the potential confounding effects of medication, a sensitivity analysis was performed excluding patients receiving anti-TNF biologics. Additionally, partial correlations between medication duration and fixel metrics were examined. Exploratory subgroup comparisons were corrected for multiple testing using the Benjamini-Hochberg FDR method.

## Supplementary Information

Below is the link to the electronic supplementary material.

Supplementary Material 1

## Data Availability

All data supporting the findings of this study are available upon request to the corresponding author and with the consent of all authors.
