# Supplementary material for: Fixel-based analysis reveals whole-brain white matter microstructural alterations in axial spondyloarthritis
Source: Sci Rep. 2026 Mar 20;16:14271. doi: 10.1038/s41598-026-45157-1 (PMC13139427; doi:10.1038/s41598-026-45157-1)
Supplement: Supplementary file 1 — Supplementary Material 1 [file 41598_2026_45157_MOESM1_ESM.docx]

| **Tabel S1.** Comparisons of White Matter Metric between Untreated axSpA patients and HCs | | | | | | | |
| --- | --- | --- | --- | --- | --- | --- | --- |
| Metrics | Untreated axSpA group | HCs | t value | P | P_FDR_ | Cohen'd | 95%CI |
|  | n=16  (mean, SD) | n=41  (mean, SD) |  |  |  |  |  |
| logFC(left EC) | 0.021(0.065) | -0.031(0.078) | 2.341 | 0.023 | 0.035 | 0.690 | 0.095-1.279 |
| FDC(right EC) | 0.684(0.081) | 0.637(0.074) | 2.095 | 0.041 | 0.041 | 0.618 | 0.026-1.204 |
| FDC(right UF) | 0.743(0.090) | 0.676(0.098) | 2.473 | 0.019 | 0.035 | 0.701 | 0.106-1.290 |
| Note: Generalized linear models were used for analysis, with age, sex and education level as control variables. axSpA: axial spondyloarthritis; HC: healthy controls; EC: external capsule; UF: uncinate fasciculus; FC: fiber bundle cross-section; FDC: fiber density and cross-section. | | | | | | | |
